# Impact of Hospitalization on the Quality of Life of Patients with Chronic Kidney Disease in Saudi Arabia

**DOI:** 10.3390/ijerph19159718

**Published:** 2022-08-07

**Authors:** Sahbanathul Missiriya Jalal, Mini Rani Mary Beth, Zahra Mohammed Bo Khamseen

**Affiliations:** 1 Department of Nursing, College of Applied Medical Sciences, King Faisal University, Al-Ahsa 31982, Saudi Arabia; 2 King Faisal Hospital, Ali Ibn Abi Talib, Al Fadhliyah, Bishah 36361, Saudi Arabia

**Keywords:** quality of life, hospitalization, CKD, chronic illness, mental health, patients, hemodialysis

## Abstract

Chronic kidney disease (CKD) is a global health problem. CKD causes patients to be hospitalized for a longer time to treat the disease. The impact of the hospitalization brings major changes and affects the quality of life (QoL) of the patients. In this study, we aimed to evaluate the impact of hospitalization on the QoL of patients with CKD. A cross-sectional study was conducted at the inpatient departments (IPDs) and outpatient departments (OPDs) of selected hospitals, in the eastern region of Saudi Arabia. The kidney disease quality of life (KDQOL) scale was used for the data collection and the findings were analyzed. The hospitalized patients had a poorer QoL than the OPD patients. The physical component summary (PCS) and mental component summary (MCS) mean scores were 52.82 ± 2.32 and 52.57 ± 2.93 in IPD patients, respectively, and 63.46 ± 3.65 and 66.39 ± 0.91 in OPD patients, respectively, which was significant (*p* < 0.0001). The QoL of patients decreased in the end stages of CKD. A significant association was observed between gender, occupation, smoking, and the stages of CKD with the QoL of the hospitalized patients. Measures must be taken to improve the QoL of these patients at all levels.

## 1. Introduction

Chronic kidney disease (CKD) is a long-term illness and is a major global public health problem [1]. In 2010, approximately 2.62 million people required treatments such as dialysis due to CKD; the need for dialysis is projected to double by 2030 [2,3]. The expenditure on treatments for kidney disease and its complications is high. The incidence and prevalence of patients with CKD are increasing worldwide. CKD has also become a debilitating problem in Saudi Arabia [4].

One of the most effective treatments for patients with CKD is hemodialysis (HD) [5]. Peritoneal dialysis and kidney transplantation are other common alternative treatments [6]. The number of patients attending HD sessions is rapidly increasing, with an estimated annual increase of 8.6% [7,8]. Adults with CKD have the highest hospitalization rate; even moderate reductions in kidney functions have been associated with elevated rates of the hospitalization [9]. Living with these types of significant challenges and restrictions may impair normal physical and psychosocial development of individuals, particularly those who are hospitalized [10,11]. As this disease is often asymptomatic at the initial stage, the awareness of CKD is low in the general population. People unexpectedly diagnosed with CKD may struggle to adjust their lives [12,13].

Quality of life (QoL) is an important criterion that illustrates the effectiveness of healthcare as well as the health level and wellbeing. QoL—or specifically, health-related quality of life (HRQOL)—is the perception of people or patients regarding their own physical, mental, and social health that may be influenced by the diagnosis, treatment, follow-up care, and duration of survival. This can be assessed using a well-validated tool or instrument [14,15,16]. According to the World Health Organization (WHO), QoL has a direct impact on physical, mental, and social health as well as emotions as it is a multi-dimensional view [17].

CKD patients can spend many hours of their lives in hospitals. Such constraints affect the living conditions of these patients and ultimately influence their quality of life [18]. CKD leads to many health consequences, including an increased risk of cardiovascular morbidity, prolonged hospitalization, a shortened lifespan, long-term care, or premature mortality and has a severe impact on the QoL of patients [19]. Patients with CKD may also be affected by other co-morbidities such as diabetes mellitus (DM), hypertension (HT), kidney infections, and cardiac problems.

A poor QoL can be a major constraint in the lives of CKD patients; its occurrence may worsen the course of the disease [20,21,22]. Considering these factors and the importance of QoL on the personal and social lives of patients with CKD, the objectives of the present study were to determine the quality of life of CKD patients in Saudi Arabia due to the impact of hospitalization and to provide health promotion to those patients.

## 2. Materials and Methods

### 2.1. Study Design

A descriptive design-based cross-sectional study was conducted among patients with CKD from January 2022 to April 2022 at the inpatient departments (IPDs) and outpatient departments (OPDs) of nephrology units in governmental and non-governmental hospitals in the eastern province of the Kingdom of Saudi Arabia to assess the impact of hospitalization. The study protocol was approved by the Institutional Review Board, King Faisal University, Hofuf, Saudi Arabia. The objectives and the procedures of the study were fully understood by the participants through the provision of clear information. All participants provided informed consent for inclusion in the study.

### 2.2. Sample Size and Sampling Method

The sample size for this study was estimated according to the consideration of the mean and standard deviation of a similar, previous study [23] with an α error of 0.05 and a β error of 0.20. A power analysis was used to estimate the required sample size. This was a two-sided *t*-test with an equal number of samples in two groups. Accounting for a 10% dropout rate, the total required sample size was calculated as 176, which was rounded up to 180 for the study. Hence, a total of 180 patients with CKD, including 90 patients from OPDs and 90 patients who were admitted to IPDs such as renal wards, were selected by a randomization technique to determine the impact of hospitalization on their QoL.

### 2.3. Inclusion Criteria

The inclusion criteria for this study were: patients with CKD at any stage from 1 to 5; who had been diagnosed with CKD for a minimum of one year or more; who were aged 20 or above, including both male and female genders; who visited a kidney OPD or were admitted to the renal wards of government or private hospitals in the eastern province of Saudi Arabia for longer than a month; and who were on a treatment such as HD, an immune suppressant therapy, or any other treatment. Patients who voluntarily provided informed consent and who were available during the data collection were included in the study. Unconscious or semi-conscious patients were excluded from this study.

### 2.4. Data Collection Tool and Procedure

The participants were interviewed to complete a structured questionnaire, which included demographic and clinical information as well as a KDQOL-SF scale with 36 items. The tool used was developed in English and the time ranged from approximately 20 to 30 min to collect the data. The study was piloted to test the reliability of the questionnaire (r = 0.736) using Cronbach’s alpha with a 95% confidence interval. The study purposes were clearly explained to the patients prior to the data collection. The collected data were encrypted and stored in a secure place to ensure privacy and confidentiality of the details of the participants. Written informed consent was obtained from each participant before the data collection.

#### 2.4.1. Demographic Information

The first part of the structured tool was the collection of the demographic information of the patients with CKD, including age, gender, educational level, occupational status, marital status, smoking status, and family history of CKD.

#### 2.4.2. Clinical Information

The second part of the structured tool was the clinical information of the patients with CKD, including co-morbid conditions such as DM, HT, heart disease, other disease conditions, as well as the stage of CKD, duration of kidney disease (at least one year after diagnosis), and type of treatment for the kidney disease.

#### 2.4.3. KDQOL

We used the KDQOL-SF tool [24,25], which consists of 36 items. This instrument is divided into eight different aspects: physical functioning; the role of physical function; body pain; general health; emotional wellbeing; the role of emotional function; social function; and energy or fatigue. The results of each scale vary from 0 to 100 (worst to best possible status). The physical and mental aspects of the eight scales are combined into a physical component summary (PCS) and a mental component summary (MCS). This tool has been widely used in studies on patients with CKD.

### 2.5. Ethical Considerations

This research was ethically approved by the Postgraduate and Scientific Research Committee, Deanship of Scientific Research, King Faisal University. The study followed the ethical principles according to the Declaration of Helsinki. Informed consent was obtained from the patients prior to the collection of the data. Participants were informed that participation in the research was voluntary, and they could withdraw from the study at any time. The privacy, confidentiality, and anonymity of the patients were assured.

### 2.6. Statistical Analysis

Statistical Package for Social Sciences (SPSS) Version 21.0 (Armonk, New York, NY, USA: International Business Machines (IBM) Corporation) was used to analyze the study results. The normality of each quantitative variable was examined for its consistency and coherency. The assumption of normality was evaluated by a Kolmogorov–Smirnov test. Numbers and percentages were given in the form of a frequency distribution for the categorical variables and the mean ± standard deviation (SD) with a 95% confidence interval was provided for the continuous variables in the descriptive statistics. Inferential statistics, using Pearson’s chi-squared or Fisher’s exact tests were used to find the homogeneity of the demographic and clinical information between the IPD and OPD patients. A Mann–Whitney U test for skewed data was applied for the comparisons between the two groups of patients from IPDs and OPDs. A one-way ANOVA was used to associate the QoL with the demographic and clinical information of the patients. All the tests of significance were two-tailed with a *p*-value < 0.05 indicating a statistically significant difference and <0.001 showing a highly significant difference.

## 3. Results

### 3.1. Demographic Information of the Patients

Out of the 180 patients included in the analysis, 75 (41.7%) were aged 41–60 years old and 79 (43.9%) were over 60 years of age (Table 1). The majority (111; 61.7%) were male. Most of them (62; 34.4%) studied at a higher secondary education level and 61 (33.9%) had a high-school level as their educational qualification. Regarding the occupational status of the patients, 63 (35%) were unemployed and 54 (30%) were employed. A total of 168 participants (93.3%) were married. Regarding the smoking status, 91 (50.6%) smoked. A total of 25 (13.9%) patients had a family history of CKD. There were no significant differences in the demographic information of the patients between the IPD and OPD groups, which demonstrated the homogeneity of the samples.

### 3.2. Clinical Information of the Patients

The clinical information indicated that out of 180 patients, 65 (36.1%) had HT as a co-morbidity (Table 2). Many of them (51; 28.3%) were at stage 3; 49 (27.2%) were at stage 2, and 33 (18.3%) were at stage 4. A total of 53 (29.1%) participants had endured CKD for a duration of approximately 4 to 6 years. Most of the patients with CKD (74; 41.1%) received immunosuppressants as a treatment for CKD and 54 (30%) received HD as a treatment.

### 3.3. KDQOL Score

The score of the KDQOL is shown in Table 3. The mean value and SD in the IPD and OPD groups, respectively, were physical function 53.75 ± 13.69 and 63.62 ± 12.04; physical role 53.38 ± 11.78 and 63.98 ± 11.72; body pain 50.12 ± 11.41 and 57.61 ± 13.37; and general health 55.92 ± 11.88 and 64.39 ± 10.48. The U test score proved the statistical significance (*p* < 0.0001). The mean value and SD in the IPD and OPD groups, respectively, were emotional wellbeing 51.23 ± 11.55 and 66.95 ± 10.27; emotional role 55.93 ± 11.58 and 65.34 ± 11.09; social function 50.56 ± 9.36 and 66.87 ± 9.9; and energy/fatigue 50.94 ± 11.14 and 67.68 ± 9.02. These results were also highly significant (*p* < 0.0001). The summary score of the KDQOL-SF scale is given in Table 4; the PCS mean score was 52.82 ± 2.32 in IPD patients and 63.46 ± 3.65 in OPD patients. The U test result was 7.1123, which was significant (*p* < 0.0001). The total score of QoL was lower among IPD patients than OPD patients in all aspects. Regarding the MCS, the mean score was 52.57 ± 2.93 in IPD patients and 66.39 ± 0.91 in OPD patients; this was also significant (*p* < 0.0001). The total U test score was 8.14796, which also showed a high-significance difference between the IPD and OPD patients (*p* < 0.0001). The overall QoL was higher in OPD patients than in IPD patients, with a significance of *p* < 0.0001.

### 3.4. Association of Variables with KDQOL-SF Summary Score

There was a significant association found between the demographic information such as gender, occupation, and smoking habit (*p* < 0.0001) in the summary scores of the KDQOL of both the IPD and OPD patients (Table 5). A significant association was also found between age, educational status, and marital status with the KDQOL summary scores (*p* < 0.0001) and a family history of CKD (*p* < 0.0113) in OPD patients. Regarding the clinical variables, there was a significant association found for the stage of CKD in the summary scores of the KDQOL for both IPD and OPD patients (*p* < 0.0001) (Table 6). The association between the duration of CKD and the treatment of CKD resulted in summary scores of the KDQOL of *p* < 0.0001 for the IPD patients. In the OPD patients, an association was found with the summary scores of the KDQOL between co-morbidities (*p* < 0.0186) and the treatment of CKD (*p* < 0.0001).

## 4. Discussion

The KDQOL tool was used to assess the impact of hospitalization on the QoL of CKD patients. The overall results demonstrated that the hospitalized patients had a poorer QoL than the OPD patients. Along with the treatment, counselling should be regularly given to IPD patients to improve their QoL. Most of the patients were male (61.7%), which supported research undertaken in Pakistan [26]. The overall mean score was 64.56 ± 3.18 in the OPD patients and 52.73 ± 2.36 in the IPD patients, with a significance at *p* < 0.0001. The PCS mean score was significantly lower (63.46 ± 3.65) than the MCS mean value (66.39 ± 0.91) in the OPD patients. The PCS score (52.82 ± 2.32) was higher and significantly different from the MCS mean score (52.57 ± 2.93) in the IPD patients. This supported the results of a study conducted on the QoL of patients with moderate to advanced chronic kidney disease in Ghana, which found that the MCS score was significantly lower than the PCS score [27]. In another study, there was no significant difference found between the mean PCS, MCS, and the Karnofsky performance status scores when the patients were divided by the etiology of CKD and the occurrence of hospitalization [28].

Regarding the specific domain of the KDQOL, the QoL of hospitalized patients was lower than that of the OPD patients. The pain score was poorer than the other domains of the KDQOL tool in this study. A previous study was undertaken to assess QoL and correlate it with musculoskeletal problems. It reported that most of the patients had either one or more musculoskeletal problems such as muscle cramps, myalgias, and arthralgias. In another study, depression was the second most common accompanying disease to CKD [29,30,31]. In this study, we observed that the mean value of emotional wellbeing indicated that the hospitalized patients had a poorer QoL than the OPD patients.

The association of the demographic variables with the KDQOL demonstrated that an increase in the age of the patients decreased their QoL; this was a significant association in OPD patients in this study (*p* < 0.0001). It has also been identified that older patients have much poorer physical functioning scores [32,33]. Considering the gender variable, male patients had a lower QoL than the female patients in both the IPD and OPD groups; this was highly significant (*p* < 0.0001). A few researchers have observed a lower level of QoL in women compared with men who were affected by chronic conditions such as asthma [34]. However, several studies did not show any differences in QoL in relation to sex [35,36,37]. In our study, the retired patients had a poorer QoL than the working patients. There was also a significant association found for the smoking status of CKD patients and their QoL (*p* < 0.0001). Research has proven that smoking is associated with an increased risk of kidney failure [38]. Heavy smokers had a significantly lower QoL score than moderate and light smokers [39].

In this study, we observed that the greatest number of patients with CKD had co-morbidities such as HT (36.1%) and DM (29.4%). Other research undertaken on QoL among CKD patients demonstrated that most participants had chronic glomerulonephritis, followed by DM and HT [27,40]. In another study, the evidence showed a higher prevalence of heart failure among patients who received HD [41]. The mean score of the QoL of patients with CKD in Iran was indicated to be greater than that of patients with heart disease, diabetic patients, and patients with cancer [42,43].

The present study revealed that the QoL decreased across all CKD stages, which was similar to other study findings. The QoL gradually decreased at each stage of CKD; we observed that the mean score in the first stage was 69.46 ± 5.58; the second stage was 66.38 ± 4.64; the third stage was 53.71 ± 3.31; the fourth stage was 45.46 ± 3.25; and the fifth stage was 39.44 ± 1.86, which was highly significant (*p* < 0.0001) in the hospitalized patients. These results supported those of other studies in which there was a lower QoL across all stages of CKD [44,45]. In this study, we observed that the QoL decreased as the duration of CKD increased, e.g., from 1 to 3 years of illness, the mean value was 71.74 ± 2.63 and for 10 years and above the score reduced to 41.34 ± 2.87, which also showed a high significance (*p* < 0.0001) among the IPD patients. In other studies, regarding HD duration, there were also no differences observed between those who were below or above 36 months of treatment [46,47].

In our study, the treatment modalities of CKD were significantly associated with the QoL of patients (*p* < 0.0001). This supported the findings of other studies [48,49]. In a few studies, the findings showed that the QoL for patients with CKD was higher than in patients on dialysis, but lower than in kidney transplant recipients [50,51].

This study had a few limitations. Although we were able to associate many factors with the QoL of CKD patients, we could not follow up the patients further and could not identify the factors worsening the conditions due to the cross-sectional design. The study design also did not allow for the inclusion of the sickest patients, who might have had a worse QoL. Patients were interviewed rather than self-selected to answer the questionnaire. We were not able to discover the cause of a low QoL from the perspective of the patients; this could have been investigated through focus group discussions. This study could be replicated as a qualitative study with in-depth interviews or as a prospective study.

## 5. Conclusions

In this study, we observed that QoL was impaired across the five CKD stages. The mean score of the QoL for patients with CKD was lower in the hospitalized patients when compared with the OPD patients. Those patients with a longer duration of CKD had a lower QoL in the hospitalized patients. There was a statistically significant association found between gender, occupation, smoking, and the stage of CKD (*p* < 0.0001) with the KDQOL in both IPD and OPD patients. Therefore, necessary measures such as counselling therapy should be provided to improve the QoL of patients at all levels to help them to avoid hospitalization and to reduce the cost of healthcare treatments.

## Figures and Tables

**Table 1:** Demographic information of the patients.

Variables		Total (*n* = 180)	IPD (*n* = 90)	OPD (*n* = 90)	*p* Value
		No. (%)	No. (%)	No. (%)	
Age (years)	20–40 years	26 (14.4)	12 (13.3)	14 (15.6)	
	41–60 years	75 (41.7)	34 (37.8)	41 (45.6)	*p* = 0.4
	More than 60 years	79 (43.9)	44 (48.9)	35 (38.9)	
Gender	Male	111 (61.7)	53 (58.9)	58 (64.4)	*p* = 0.44
	Female	69 (38.3)	37 (41.1)	32 (35.6)	
Educational Level	Primary	20 (11.1)	12 (13.3)	8 (8.9)	
	High school	61 (33.9)	29 (32.2)	32 (35.6)	*p* = 0.41
	Higher secondary	62 (34.4)	34 (37.8)	28 (31.1)	
	College	37 (20.6)	15 (16.7)	22 (24.4)	
Occupation	Employed	51 (28.3)	25 (27.8)	26 (28.9)	
	Business	54 (30)	32 (35.6)	22 (24.4)	*p* = 0.38
	Retired	22 (12.2)	9 (10)	13 (14.4)	
	Unemployed	63 (35)	24 (26.7)	29 (32.2)	
Marital Status	Married	168 (93.3)	85 (94.4)	83 (92.2)	*p* = 0.55
	Unmarried	12 (6.7)	5 (5.6)	7 (7.8)	
Smoking Habit	Yes	91 (50.6)	43 (47.8)	48 (53.3)	*p* = 0.46
	No	89 (49.4)	47 (52.2)	42 (46.7)	
Family History of CKD	Yes	25 (13.9)	9 (10)	16 (17.8)	*p* = 0.13
	No	155 (86.1)	81 (90)	74 (82.2)	

No—number; %—percentage; statistically significant at *p* < 0.05.

**Table 2:** Clinical information of the patients.

Variables		Total (*n* = 180)	IPD (*n* = 90)	OPD (*n* = 90)	*p* Value
		No. (%)	No. (%)	No. (%)	
Co-morbidities	DM	53 (29.4)	29 (32.2)	24 (26.7)	
	HT	65 (36.1)	34 (37.8)	31 (34.4)	*p* = 0.33
	Heart disease	29 (16.1)	10 (11.1)	19 (21.1)	
	Others	33 (18.3)	17 (18.9)	16 (17.8)	
Stage of CKD	1	36 (20)	3 (3.3)	33 (36.7)	
	2	49 (27.2)	17 (18.9)	32 (35.6)	
	3	51 (28.3)	34 (37.8)	17 (18.9)	*p* = 0.001
	4	33 (18.3)	27 (30)	6 (6.7)	
	5	11 (6.1)	9 (10)	2 (3)	
Duration of CKD	1–3 years	38 (21.1)	8 (8.9)	38 (42.3)	
	4–6 years	53 (29.1)	29 (32.2)	24 (26.7)	*p* = 0.001
	7–9 years	54 (30)	38 (42.2)	16 (17.8)	
	10 and above years	27 (15)	15 (16.7)	12 (13.3)	
Treatment of CKD	HD	54 (30)	38 (42.2)	16 (17.8)	
	Immunosuppressants	74 (41.1)	32 (35.6)	42 (46.7)	*p* = 0.001
	Others	52 (28.9)	20 (22.2)	32 (35.6)	

No—number; %—percentage; statistically significant at *p* < 0.05.

**Table 3:** KDQOL-SF scores of the patients (*n* = 180).

Dimensions	Number of Items	Total Patients (*n* = 180) Mean ± SD	IPD Patients (*n* = 90) Mean ± SD	OPD Patients (*n* = 90) Mean ± SD	’U’ Test	*p* Value
Physical function	10	58.69 ± 13.77	53.75 ± 13.69	63.62 ± 12.04	5.09963	0.0001 *
Role physical	4	58.68 ± 12.87	53.38 ± 11.78	63.98 ± 11.72	5.60315	0.0001 *
Body pain	2	53.87 ± 12.95	50.12 ± 11.41	57.61 ± 13.37	3.9295	0.0001 *
General health	5	60.16 ± 11.95	55.92 ± 11.88	64.39 ± 10.48	4.72913	0.0001 *
Emotional wellbeing	5	59.09 ± 13.45	51.23 ± 11.55	66.95 ± 10.27	7.93196	0.0001 *
Role emotional	3	60.64 ± 12.25	55.93 ± 11.58	65.34 ± 11.09	5.14111	0.0001 *
Social function	2	58.71 ± 12.62	50.56 ± 9.36	66.87 ± 9.9	8.68582	0.0001 *
Energy/fatigue	4	59.31 ± 13.13	50.94 ± 11.14	67.68 ± 9.02	8.75877	0.0001 *

* Statistically significant at *p* < 0.05.

**Table 4:** KDQOL-SF summary scores of the patients (*n* = 180).

Scales	Total Patients (*n* = 180) Mean ± SD	IPD Patients (*n* = 90) Mean ± SD	OPD Patients (*n* = 90) Mean ± SD	’U’ Test	*p* Value
PCS	58.14 ± 2.46	52.82 ± 2.32	63.46 ± 3.65	7.1123	0.0001 *
MCS	59.48 ± 1.02	52.57 ± 2.93	66.39 ± 0.91	8.7087	0.0001 *
Total Score	58.64 ± 2.06	52.73 ± 2.36	64.56 ± 3.18	8.14796	0.0001 *

* Significant difference at *p* < 0.05; highly significant difference at *p* < 0.001.

**Table 5:** Association of demographic variables with KDQOL-SF summary scores of the patients.

Variables		IPD (*n* = 90)	*p* Value	OPD (*n* = 90)	*p* Value
		Mean ± SD		Mean ± SD	
Age (years)	20–40 years	54.5 ± 12.34	0.2139 NS	72.55 ± 1.66	0.0001 *
	41–60 years	54.45 ± 7.9		65.58 ± 2.03	
	More than 60 years	50.92 ± 9.77		60.16 ± 3.35	
Gender	Male	47.57 ± 5.74	0.0001 *	62.63 ± 4.09	0.0001 *
	Female	60.11 ± 9.11		68.04 ± 4.54	
Educational Level	Primary	52.65 ± 12.74	0.1335 NS	57.13 ± 2.46	0.0001*
	High school	53.11 ± 10.56		62.2 ± 3.35	
	Higher secondary	54.65 ± 7.99		68.35 ± 3.15	
	College	47.70 ± 6.54		65.86 ± 4.78	
Occupation	Employed	54.08 ± 7.37		65.78 ± 2.71	0.0001 *
	Business	48.03 ± 6.66		63.17 ± 3.63	
	Retired	47.59 ± 6.42	0.0001 *	58.46 ± 3.01	
	Unemployed	59.51 ± 11.47		67.25 ± 5.51	
Marital Status	Yes	53.07 ± 9.4	0.167 NS	63.97 ± 4.72	0.0001 *
	No	48.97 ± 11.42		71.5 ± 0.99	
Smoking	Yes	46.86 ± 5.79	0.0001 *	61.51 ± 3.32	0.0001 *
	No	58.1 ± 9.19		68.04 ± 4.2	
Family History of CKD	Yes	51.51 ± 12.93	0.6888 NS	61.73 ± 5.84	0.0113 *
	No	52.86 ± 9.2		65.17 ± 4.58	

* Significant at *p* < 0.05; highly significant at *p* < 0.001; NS—non-significant.

**Table 6:** Association of clinical variables with KDQOL-SF summary scores of the patients.

Variables		IPD (*n* = 90)	*p* Value	OPD (*n* = 90)	*p* Value
		Mean ± SD		Mean ± SD	
Co-morbidities	DM	55.45 ± 8.55	0.2688 NS	65.91 ± 4.89	0.0186 *
	HT	50.71 ± 10.05		63.81 ± 3.55	
	Heart disease	52.78 ± 10.2		66.35 ± 5.86	
	None	52.09 ± 9.52		61.85 ± 5.23	
Stage of CKD	1	69.46 ± 5.58		69.61 ± 3.02	0.0001 *
	2	66.38 ± 4.64		63.09 ± 1.78	
	3	53.71 ± 3.31	0.0001 *	61.46 ± 2.83	
	4	45.46 ± 3.25		57.1 ± 2.09	
	5	39.44 ± 1.86		53.37 ± 0.83	
Duration of CKD	1–3 years	71.74 ± 2.63	0.0001 *	67.94 ± 3.62	
	4–6 years	59.31 ± 4.17		65.26 ± 3.43	
	7–9 years	48.2 ± 4.04		63.66 ± 5.81	0.1086
	10 and above years	41.34 ± 2.87		63.65 ± 5.19	NS
Treatment of CKD	Hemodialysis	44.02 ± 3.82	0.0001 *	62.17 ± 7.28	
	Immunosuppressants	54.53 ± 2.69		63.45 ± 3.41	0.0004 *
	Others	66.4 ± 5.78		67.2 ± 4.24	

* Statistically significant at *p* < 0.05; NS—non-significant.

## Data Availability

Not applicable.

## Abbreviations

CKD	Chronic Kidney Disease
HD	Hemodialysis
IBM	International Business Machines Corporation
IPD	Inpatient Department
KDQOL-SF	Kidney Disease Quality of Life Short Form
MCS	Mental Component Summary
OPD	Outpatient Department
PCS	Physical Component Summary
QoL	Quality of Life
SD	Standard Deviation
SPSS	Statistical Package for the Social Sciences
WHO	World Health Organization
